# The functional potential and active populations of the pit mud microbiome for the production of Chinese strong‐flavour liquor

**DOI:** 10.1111/1751-7915.12729

**Published:** 2017-07-13

**Authors:** Yong Tao, Xiang Wang, Xiangzhen Li, Na Wei, Hong Jin, Zhancheng Xu, Qinglan Tang, Xiaoyu Zhu

**Affiliations:** ^1^ Key Laboratory of Environmental and Applied Microbiology Chinese Academy of Sciences & Environmental Microbiology Key Laboratory of Sichuan Province Chengdu Institute of Biology Chinese Academy of Sciences Sichuan 610041 China; ^2^ The National‐recognized Enterprise Technology Center Sichuan Jiannanchun Group Co. Ltd. Mianzhu Sichuan 618200 China; ^3^ Chengdu Medical College Chengdu 610083 China

## Abstract

The popular distilled Chinese strong‐flavour liquor (CSFL) is produced by solid fermentation in the ground pit. Microbes inhabiting in the pit mud (PM) on the walls of the fermentation pit are responsible for the production of caproic acid (CA) that determines the quality of CSFL to a large degree. However, little is known about the active microbial populations and metabolic potential of the PM microbiome. Here, we investigated the overall metabolic features of the PM microbiome and its active microbial components by combining metagenomics and MiSeq‐sequencing analyses of the 16S rRNA genes from DNA and RNA (cDNA). Results showed that prokaryotes were predominant populations in the PM microbiome, accounting for 95.3% of total metagenomic reads, while eukaryotic abundance was only 1.8%. The dominant prokaryotic phyla were *Firmicutes, Euryarchaeota, Bacteroidetes, Actinobacteria* and *Proteobacteria*, accounting for 48.0%, 19.0%, 13.5%, 2.5% and 2.1% of total metagenomic reads respectively. Most genes encoding putative metabolic pathways responsible for the putative CA production via chain elongation pathway were detected. This indicated that the PM microbiome owned functional potential for synthesizing CA from ethanol or lactate. Some key genes encoding enzymes involved in hydrogenotrophic and acetoclastic methanogenesis pathways were detected in the PM metagenome, suggesting the possible occurrence of interspecies hydrogen transfer between CA‐producing bacteria and methanogens. The 16S rDNA and 16S rRNA profiles showed that the *Clostridial* cluster IV, *Lactobacillus*,* Caloramator*,* Clostridium*,* Sedimentibacter*,* Bacteroides* and *Porphyromonas* were active populations in situ, in which *Clostridial* cluster IV and *Clostridium* were likely involved in the CA production. This study improved our understandings on the active populations and metabolic pathways of the PM microbiome involved in the CA synthesis in the CSFL fermentation.

## Introduction

Chinese strong‐flavour liquor (CSFL) is a popular distilled alcoholic beverage in China due to its distinct aroma and good taste. It accounts for about 70% market share in total liquor production in China (Zhao *et al*., 2012; Hu *et al*., 2015). The CSFL is fermented from carbohydrate‐rich raw material mixture by a unique solid‐state anaerobic fermentation process (Tao *et al*., 2014), in which the key micro‐organisms contributing fermentation are usually from the Daqu and the pit mud (PM). The former is a traditional starter culture consisting of fungi and yeasts that are responsible for simultaneous saccharification and alcoholic fermentation of the raw materials (Hu *et al*., 2015). The latter is a kind of specific fermentation mud (about 10–15 cm in thickness), also called pit mud, covered to the inside walls of a cuboid‐shaped pit (about 2 × 3 × 2 m) in the ground that is the unique fermentation vessel for the CSFL (Fig. S1).

For the fermentation procedure, the mixture of raw grain materials including wheat, sorghum, corn with the Daqu‐starter is placed into the fermentation pit. The pit is sealed with common mud, and fermentation is allowed to proceed for 60–70 days. Afterwards, fermented material is then taken out of the pit and distilled to make the CSFL. The process described above is periodically repeated in the same pit. The long‐aged pits are generally continuously used for decades, and thus, a unique microbiological flora develops in the pit mud (Tao *et al*., 2014). Microbes inhabiting in the PM produce various flavour compounds such as alcohols, fatty acids and esters. Especially, caproic acid (CA) has been identified as a key flavour substance of the CSFL. The CA‐producing bacteria are thus considered to be important functional members in the PM (Fan and Qian, 2006; Hu *et al*., 2015). .

Taxonomic studies using culture‐ and molecular‐based approaches have shown that the PM microbiota is complex and predominantly consists of the bacterial populations belonging to *Lactobacillaceae*,* Clostridiaceae*,* Ruminococcaceae*,* Porphyromonadaceae*,* Bacillaceae* and three archaeal families containing *Methanobacteriaceae*,* Methanomicrobiaceae* and *Methanosarcinaceae* (Zheng *et al*., 2013; Ding *et al*., 2014; Tao *et al*., 2014; Hu *et al*., 2015). Most micro‐organisms in the PM are obligate anaerobic bacteria, which are very difficult for cultivation, isolation and quantification due to their strict anaerobic requirements and unknown culture conditions (Doyle *et al*., 2015). Our previous studies indicated that some microbial populations in the PM, that is *Ruminococcaceae*,* Clostridiaceae* and *Lactobacillaceae,* have significant correlation with caproic acid content (Tao *et al*., 2014). Although these studies based on culture‐independent methods indicate microbial diversity of the PM microbiome, there is a lack of understanding about the functional potential of the PM microbiome and its active microbial populations in situ.

Metagenomic analyses of fermentation microbiome can reveal microbial populations and genes involved in fermentation and the production of flavouring compounds (Jung *et al*., 2011; Xie *et al*., 2013). However, microbial community based on DNA‐based distribution does not provide clues on activity of each detected bacterial population (Rodriguez‐Blanco *et al*., 2009). Bacterial growth rate is correlated with cellular rRNA content (DeLong *et al*., 1989; Poulsen *et al*., 1993); therefore, cellular activity may be obtained by tracking reverse‐transcribed 16S rRNA (cDNA). Some studies have combined rDNA‐ and rRNA‐based surveys for the simultaneous description of total and active microbial populations (Moeseneder *et al*., 2005; Rodriguez‐Blanco *et al*., 2009; Lay *et al*., 2013; Lin *et al*., 2016). In this study, we combined a metagenomic approach and MiSeq‐sequencing analyses of 16S rDNA and 16S rRNA genes to assess the PM microbiome. Our aims were to investigate metabolic potentials of the PM microbiome and to identify active microbial populations in situ that may be involved in the synthesis of CA, a key flavour substance in CSFL.

## Results

### Metagenomic sequencing statistics

In total, metagenomic sequencing resulted in 40 997 331–52 814 635 reads in PM samples, with an average length of 100 bp (Table 1). Among all sequences, the average 73.0% of total reads (63.0–80.4%) passed the quality control (QC). The numbers of reads representing predicted protein and rRNA features were 45.2–50.0% and 1.2–1.4% of valid sequences that passed QC respectively. The GC contents of the sequences in the data sets ranged from 42 to 46%. Among these predicted protein‐ and rRNA‐related reads, the average 29.1% and 1.1% could match to known protein and rRNA sequences, respectively, but a majority of predicted protein (71.5%) and rRNA (98.9%) remained unidentified due to a lack of comparable reference sequences. It highlighted the need to further isolate, characterize and sequence new strains from the PM to supplement existing databases, so that the annotation of metagenomic data from unique fermentation reactors could be more reliable and informative.

**Table 1:** Overall statistics of PM metagenome

Parameter	PM_626_	PM_663_	PM_837_
Total sequence number	40 997 331	45 004 143	52 814 635
Total sequence size (bp)	4 100 165 901	4 500 843 600	5 282 104 072
Average sequence length (bp)	100	100	100
No. of sequences that passed QC	31 885 704	34 059 404	33 294 594
No. of predicted protein features	15 420 040	15 658 630	15 054 555
No. of identified protein features	4 741 135	4 316 178	4 314 752
No. of predicted rRNA features	395 740	408 380	461 748
No. of identified rRNA features	4 987	4 358	4 495
GC content (%)	44	46	46

### Functional gene profiles of the PM Metagenome

**Figure 1:**
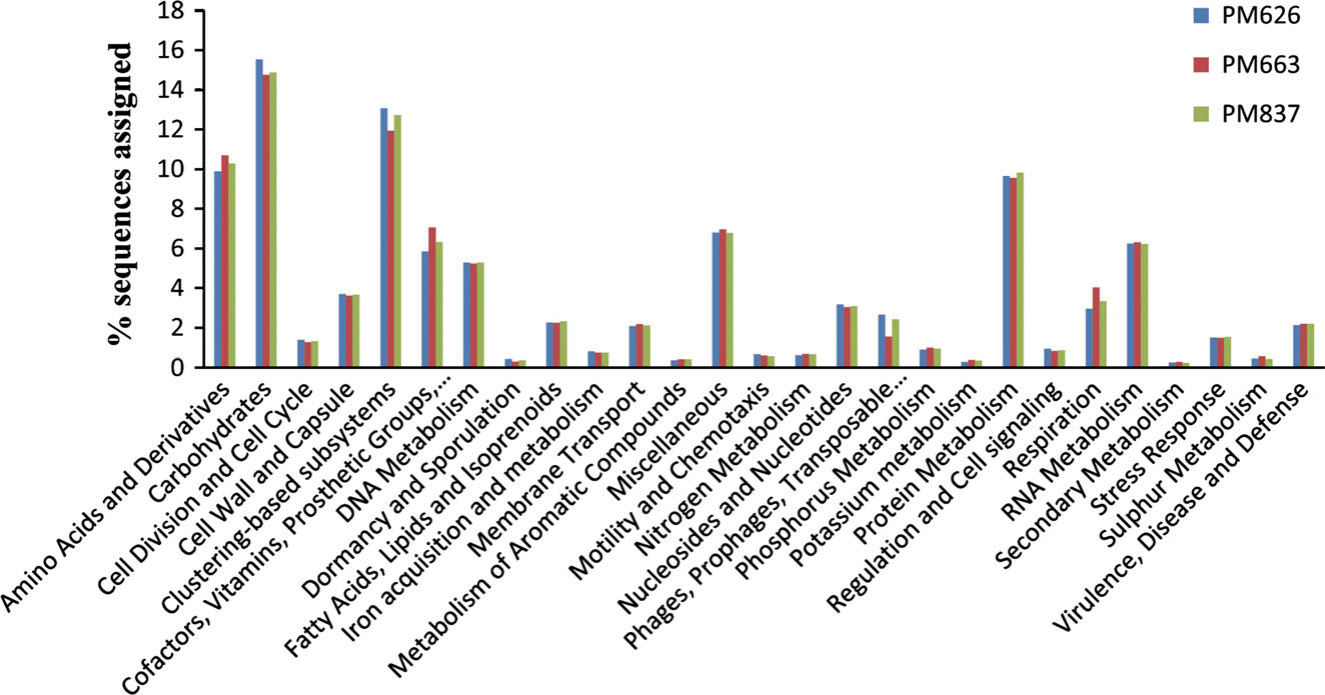
Predicted metabolic profiles of PM microbiome inferred from metagenome. The MG‐RAST platform based on SEED subsystems was used for analysing the metagenomic data sets. PM_(626_,_663_,_837)_ represents three PM samples selected from three CSFL fermentation pits respectively.

**Figure 2:**
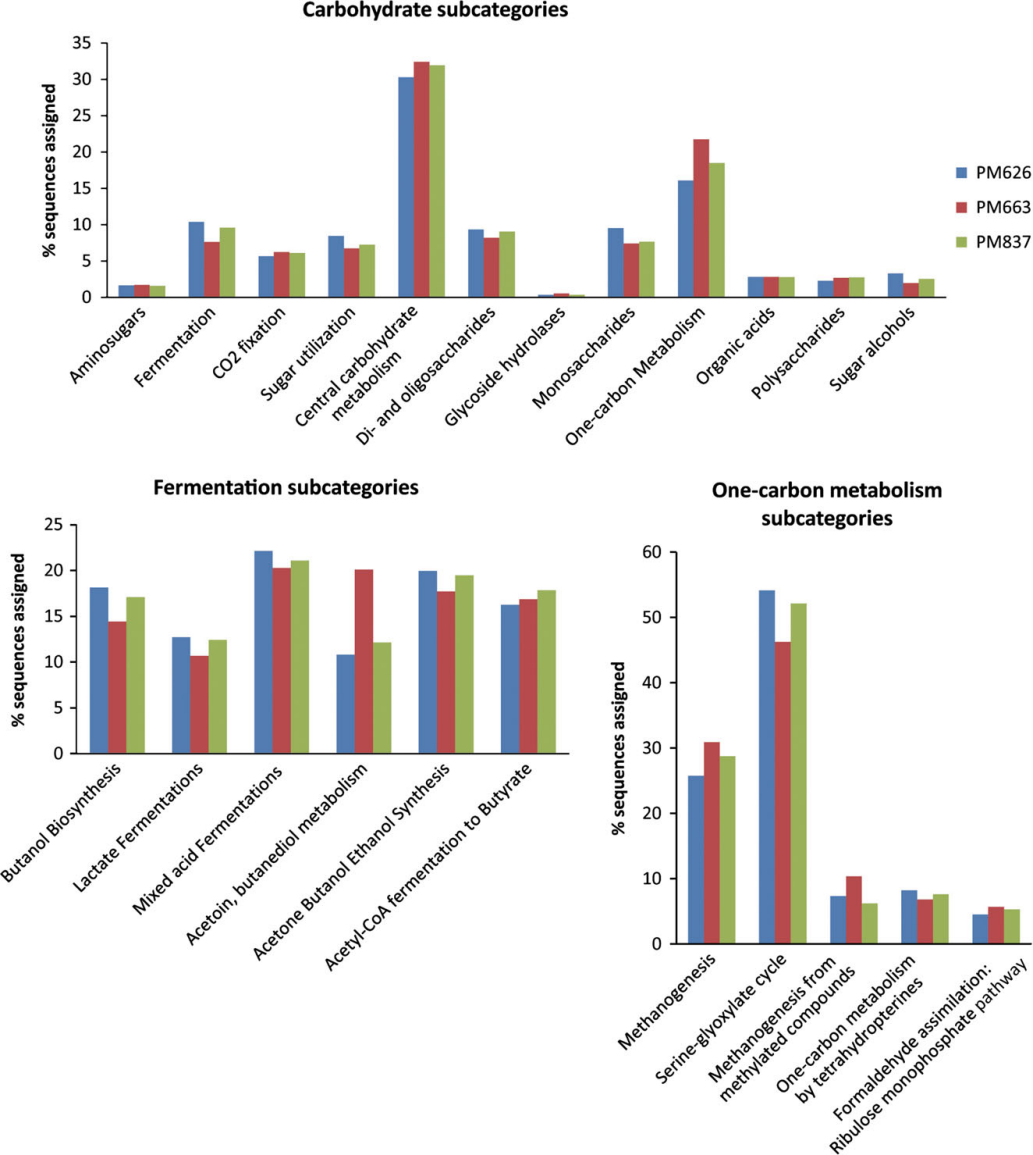
Predicted metabolic profiles of PM metagenome related to carbohydrate metabolism fermentation, and one‐carbon metabolism. The abundances of metabolic genes in the MG‐RAST server were calculated using the number of sequencing reads within each subsystem rather than using the number of total metagenomic reads.

**Figure 3:**
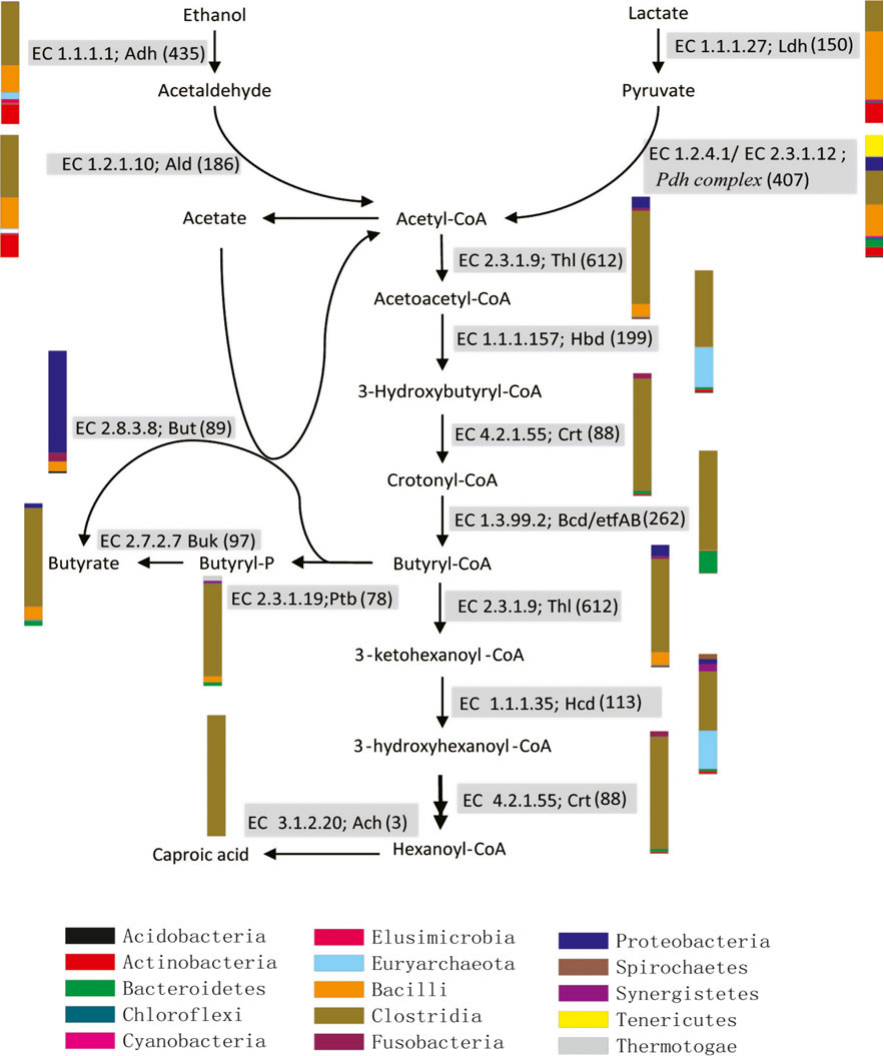
Phylogenetic profiles based on key genes encoding the key enzymes in chain elongation pathway. Colour‐coded bars indicated the relative abundances in percentage of different phylotypes for each gene category. The classification was based on phyla, except for the Firmicutes, which were shown at the class level. Each enzyme designation is preceded by the enzyme nomenclature designation and followed by the average number of reads annotated from three PM metagenomes (in parentheses).

To explore the metabolic potential of the PM microbiome, all sequences were analysed using the SEED subsystem publicly available on the MG‐RAST server. The functional gene profile at level‐1 was mainly composed of carbohydrate metabolism (14.9–15.5%), clustering‐based subsystems (12.5–13.1%), amino acid biosynthesis (9.9–10.3%), protein metabolism (9.6–9.8%), and vitamin and pigment metabolism (5.8–6.3%). DNA and RNA metabolism‐related sequences constituted 5.3% and 6.2% in average respectively (Fig. 1). The high abundances of genes related to carbohydrate metabolism were observed in PM metagenomes. In particular, abundant genes were involved in central carbohydrate metabolism and one‐carbon metabolism (Fig. 2). The central carbohydrate metabolism subcategories were mostly associated with glycolysis, gluconeogenesis and pyruvate metabolism (Fig. S2). The one‐carbon metabolism was linked with methanogenesis and serine‐glyoxylate cycle (Fig. 2). The PM microbiome is thought to be responsible for the production of various flavour compounds, especially CA, a key flavour substance in CSFL. Thus, the genes and microbial populations involved in CA production were surveyed in detail. CA is generally produced by anaerobic bacteria via chain elongation pathway coupling ethanol oxidation with reversed β‐oxidation (Fig. 3). Ethanol is one of the most efficient reduced substrate for CA synthesis. The oxidation of ethanol can provide energy (acetyl‐CoA) to sequentially elongate the carbon chain of carboxylic acids (e.g. acetic acid to *n*‐butyric acid to CA etc.). Alcohol dehydrogenase (EC 1.1.1.1, *Adh*) and acetaldehyde dehydrogenase (EC 1.2.1.10, *Ald*) are two key enzymes that convert ethanol into acetyl‐CoA. Both *Adh* and *Ald* genes were detected in PM metagenomes with relative abundances of 2010 and 2229 reads per million (RPM) respectively (Table 2).The two genes matched mostly to the genera *Clostridium*,* Lactobacillus* and *Atopobium* (Table S1). Likewise, lactate is a reduced energy‐rich compound to generate *n*‐butyrate via chain elongation. Lactate dehydrogenase (EC 1.1.1.27, *Ldh*) is involved in the oxidation and reduction between pyruvate and lactate. Pyruvate dehydrogenase complex (EC 1.2.4.1/2.3.1.12, *Pdh*‐complex) converts pyruvate into acetyl‐CoA, which is added to acetate or butyrate elongating carbon chain length with two carbons at a time. The *Ldh* gene (408 RPM) and *Pdh*‐complex (221 RPM) were detected in PM metagenomes, and they were mainly matched to the genera *Clostridium*,* Lactobacillus* and *Enterococcus*. The key enzymes performing reversed β‐oxidation, including acetyl‐CoA acetyltransferase (EC 2.3.1.9, *Thl*), 3‐hydroxybutyryl‐CoA dehydrogenase (EC 1.1.1.157, *Hbd*), 3‐hydroxybutyryl‐CoA dehydratase (EC 4.2.1.55, *Crt*) and butyryl‐CoA dehydrogenase/electron‐transferring flavoprotein complex (EC 1.3.99.2, *Bcd/etfαβ*) were all detected in PM metagenomes (Table 2), and these genes were mainly matched to the genera *Clostridium* and *Syntrophomonas* (Table S1). The final step from butyryl‐CoA to butyrate was catalysed either by butyryl‐CoA: acetate CoA transferase (EC 2.8.3.8, *But*) or butyrate kinase (EC 2.7.2.7, *Buk*) after the phosphorylation of butyryl‐CoA by phosphate butyryltransferase (EC 2.3.1.19, *Ptb*). In PM metagenomes, the *But* gene was mainly matched to the members of phylum *Proteobacteria*, such as *Escherichia* spp., *Haemophilus* spp. and *Shigella* spp, while the *Buk* gene was more matched to the members of phylum *Firmicute*, for example *Clostridium* spp., *Caldanaerobacter* spp. and *Alkaliphilus* spp. It is speculated that the pathway of extending butyric acid to CA is similar to that of acetate to butyrate with key enzymes encoded by *Thl*,* Crt*,* Hcd* and *Ach* genes (Seedorf *et al*., 2008; Machado *et al*., 2012), which were detected in the PM microbiome (Table 2 and Table S1).

**Table 2:** Abundances of important genes involved in chain elongation and methanogenesis retrieved in the PM metagenome

Function enzyme	Gene	RPM^a^(reads per million)
Chain elongation		
Alcohol dehydrogenase (EC 1.1.1.1)	*Adh*	2010 ± 553
Acetaldehyde dehydrogenase (EC 1.2.1.10)	*Ald*	2229 ± 835
Lactate dehydrogenase (EC 1.1.1.27)	*Ldh*	408 ± 189
Pyruvate dehydrogenase (EC 1.2.4.1)	*Pdh‐*complex	221 ± 36
Acetyl‐CoA acetyltransferase (EC 2.3.1.9)	*Thl*	4752 ± 518
3‐hydroxybutyryl‐CoA dehydrogenase (EC 1.1.1.157)	*Hbd*	1051 ± 152
3‐hydroxybutyryl‐CoA dehydratase (EC 4.2.1.55)	*Crt*	317 ± 11
Butyryl‐CoA dehydrogenase (EC 1.3.99.2)	*Bcd/etfαβ*	3827 ± 1319
Acetyl‐CoA: acetoacetyl‐CoA transferase (EC 2.8.3.8)	*But*	76 ± 14
Phosphate butyryltransferase (EC 2.3.1.19)	*Ptb*	319 ± 57
Butyrate Kinase (EC 2.7.2.7)	*Buk*	705 ± 93
3‐Hydroxy‐acyl‐CoA dehydrogenase (EC 1.1.1.35)	*Hcd*	975 ± 166
Acyl‐CoA hydrolase (EC 3.1.2.20)	*Ach*	4 ± 2
Methanogenesis		
Acetyl‐CoA synthetase (EC 6.2.1.1)	Acs	2679 ± 513
Acetyl‐CoA decarbonylase/synthase complex (EC 1.2.99.2)	Cdh	267 ± 128
Formyl‐*MF* dehydrogenase (EC 1.2.99.5)	Fwd	2535 ± 488
Formyl‐MF:H4MPT formyl transferase (EC 2.3.1.101)	Ftr	197 ± 28
Methenyl‐H4MPT cyclohydrolases (EC 3.5.4.27)	Mch	598 ± 101
F420‐dependent methylene‐H4MPT dehydrogenase (EC 1.5.99.9)	Mtd	426 ± 92
F420‐dependent methylene‐H4MPT reductases (EC 1.5.99.11)	Mer	238 ± 41
H4MPT S‐methyltransferase (EC:2.1.1.86)	Mtr	1045 ± 164
Methyl‐Coenzyme M reductase (2.8.4.1)	Mcr	1403 ± 203

**a.** RPM means average of the relative abundances of three PM samples with a standard deviation (*n* = 3).

Hydrogenotrophic methanogenesis is thought to strengthen the interspecies hydrogen transfer between CA‐producing bacteria and methanogens and enable CA formation thermodynamically feasible (Tao *et al*., 2014). Therefore, methane metabolism was also investigated in this study. PM metagenomic analysis indicated that methanogens mainly consisted of hydrogenotrophic *Methanoculleus*,* Methanobrevibacter*, and *Methanobacterium* and acetoclastic *Methanosarcina* (Table 3). Most functional genes encoding those enzymes directly related to methanogenesis were detected in PM metagenomes (Table 2). These included enzymes involved in acetoclastic methanogenesis pathway, such as acetyl‐CoA synthetase (EC 6.2.1.1, *Acs*), and acetyl‐CoA decarbonylase/synthase complex (EC 1.2.99.2, *Cdh*). Simultaneously, enzymes (e.g. *Cdh*,* Fwd*,* Ftr*,* Mch*,* Mtd*,* Mer*,* Mtr* and *Mcr*) involved in hydrogenotrophic methanogenesis pathway were detected in PM metagenomes. This suggested the presence of hydrogenotrophic and acetoclastic methanogenesis pathways in the PM metagenome.

**Table 3:** The relative abundances of important prokaryotic populations in the PM metagenome, 16S rDNA and rRNA libraries (% of total reads)

Phylum/genus	Metagenome	16S rDNA	16S rRNA
*Euryarchaeota* a	19.00 ± 5.76	38.56 ± 6.49	4.70 ± 4.10
*Methanobacterium* a	0.89 ± 0.86	2.22 ± 0.01	0.22 ± 0.21
*Methanobrevibacter* a	4.20 ± 4.85	12.17 ± 10.96	0.23 ± 0.10
*Methanoculleus*	7.25 ± 4.82	2.00 ± 1.48	0.32 ± 0.23
*Methanosarcina* a	5.58 ± 3.63	19.16 ± 9.07	0.32 ± 0.31
*Actinobacteria*	2.47 ± 0.82	0.64 ± 0.42	0.44 ± 0.19
*Atopobium*	1.00 ± 0.57	0.30 ± 0.10	0.54 ± 0.13
*Bacteroidetes* a	13.53 ± 3.81	20.44 ± 2.58	5.55 ± 1.48
*Bacteroides*	6.67 ± 2.42	2.32 ± 0.80	2.42 ± 0.08
*Parabacteroides*	1.26 ± 0.27	ND	ND
*Porphyromonas* a	1.55 ± 0.57	16.70 ± 3.34	2.01 ± 0.73
*Chloroflexi*	0.05 ± 0.02	0.46 ± 0.10	0.51 ± 0.04
*Firmicutes* a	48.01 ± 12.27	29.33 ± 5.07	79.47 ± 6.44
*Alkaliphilus*	0.67 ± 0.09	ND	ND
*Bacillus*	1.23 ± 0.27	0.02 ± 0.01	0.01 ± 0.01
*Blautia*	0.56 ± 0.41	ND	ND
*Caloramator*	0.11 ± 0.01	3.37 ± 1.73	6.71 ± 3.45
*Clostridium*	12.15 ± 4.60	1.94 ± 0.33	1.67 ± 0.18
*Clostridial* cluster IV^a^	3.86 ± 1.38	7.35 ± 3.09	42.96 ± 9.62
*Desulfitobacterium*	0.56 ± 0.40	ND	ND
*Desulfotomaculum*	0.92 ± 0.27	0.09 ± 0.03	0.16 ± 0.07
*Enterococcus*	0.78 ± 0.28	0.06 ± 0.02	0.04 ± 0.04
*Ethanoligenens*	0.91 ± 0.29	0.02 ± 0.01	0.09 ± 0.08
*Eubacterium*	2.65 ± 0.70	0.21 ± 0.13	0.28 ± 0.03
*Faecalibacterium*	0.98 ± 0.45	ND	ND
*Finegoldia*	0.62 ± 0.15	ND	ND
*Lactobacillus* a	3.49 ± 2.55	2.33 ± 0.76	19.60 ± 5.83
*Paenibacillus*	0.99 ± 0.22	ND	ND
*Peptoniphilus*	0.84 ± 0.12	ND	ND
*Sedimentibacter*	0.91 ± 0.31	1.85 ± 0.62	1.20 ± 0.41
*Streptococcus*	0.61 ± 0.25	ND	ND
*Syntrophomonas*	3.14 ± 1.56	3.05 ± 0.60	0.98 ± 0.14
*Tepidimicrobium*	0.06 ± 0.05	0.24 ± 0.08	0.53 ± 0.12
*Tissierella*	1.21 ± 0.40	0.36 ± 0.15	0.19 ± 0.06
*Proteobacteria*	2.07 ± 0.27	1.45 ± 0.38	2.26 ± 0.63
*Acinetobacter*	0.01 ± 0.01	0.30 ± 0.11	0.54 ± 0.12
*Synergistetes*	0.86 ± 0.63	1.09 ± 0.74	0.41 ± 0.12
*Aminobacterium*	0.59 ± 0.50	0.86 ± 0.60	0.12 ± 0.06
*Tenericutes*	0.83 ± 0.19	0.26 ± 0.04	0.04 ± 0.01

ND, not detectable.

All data are average of three PM samples ± standard deviations (*n* = 3).

a Significant differences between 16S rDNA and 16S rRNA at *P *<* *0.05 (** *P *<* *0.01) as determined by ANOVA.

### Metagenomic microbial community composition

**Figure 4:**
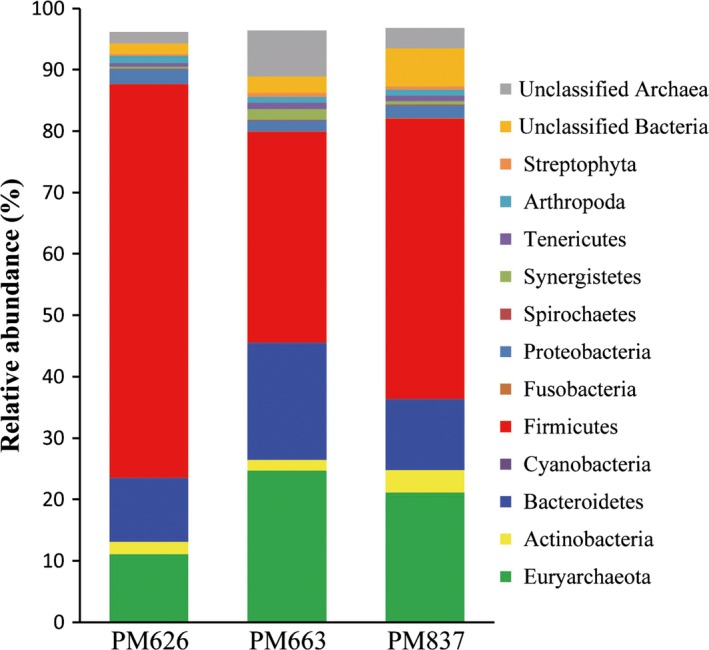
Relative abundances of different phyla in the PM metagenome. Three PM samples (PM626, PM663 and P837) were collected from three CSFL fermentation pits that had been used for 30 years.

Among PM sequences that passed the QC, 63.13–82.02%, 12.99–32.17% and 1.61–2.78% were identified as fragments originating from Bacteria, Archaea and Eukaryota respectively. The dominant bacterial phyla (> 5% of total reads) were Firmicutes (34.31–64.07%) and Bacteroidetes (10.29–18.89%). The archaeal reads were mainly affiliated to Euryarchaeota (11.12–24.71%). The small proportion of eukaryotic reads was detected in PM metagenomes, including Arthropoda (1.01–2.29%) and Streptophyta (0.22–0.65%) (Fig. 4). The results suggested that the PM microbiota were dominated by prokaryotes.

### Community profiles based on 16S rDNA and rRNA

**Figure 5:**
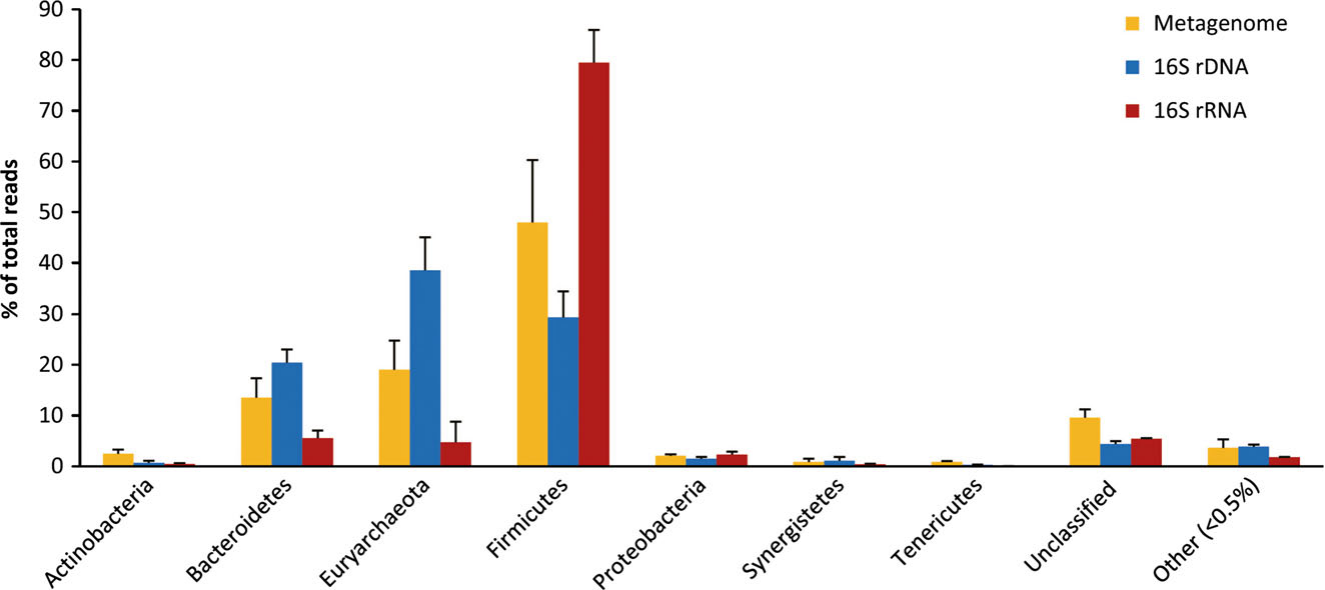
Proportions of different clades of microbial reads from PM metagenomes, 16S rDNA and 16S rRNA libraries. The classification was based on phyla, except for the Firmicutes, which were shown at the class level.

To quantify total and active microbial populations in the PM, we analysed microbial community by MiSeq‐sequencing of 16S rRNA gene from DNA and RNA (cDNA) respectively. Significant difference between microbial communities based on the 16S rDNA and the 16S rRNA was observed (Fig. 5 and Table 3). The Firmicutes was overrepresented by an over 171% increase in relative abundance in the 16S rRNA data sets (79.47%) compared to the 16S rDNA data sets (29.33%) (*P* < 0.05). In contrast, the *Bacteroidetes* and *Euryarchaeota* showed a significant decrease by 73% and 88% respectively. In 16S rRNA profiles, three dominant genera (relative abundance > 5%) were observed, including *Clostridial* cluster IV (42.96%), *Lactobacillus (*19.60%) and *Caloramator* (6.71%), but their abundances in 16S rDNA libraries were only 7.35%, 2.33% and 3.37% respectively. Other four subdominant populations with their relative abundances between 1% and 5% were *Clostridium* (1.67%), *Sedimentibacter* (1.20%), *Bacteroides* (2.42%), *Porphyromonas* (2.01%) in the 16S rRNA profiles. In total, the above seven genera accounted for more than 76.57% and 35.86% of total sequences in the 16S rRNA and rDNA libraries respectively. Meanwhile, the reads belonging to these seven genera made up 28.74% of total reads in PM metagenomes. However, some dominant genera (> 5%) in 16S rDNA libraries, such as *Methanobrevibacter* (12.17%), *Methanosarcina* (19.16%) and *Porphyromonas* (16.70%) decreased significantly in 16S rRNA libraries.

The archaeal sequences were mostly from Euryarchaeota, including methanogens such as *Methanoculleus*,* Methanosarcina* and *Methanobrevibacter*. However, the abundances of these methanogens strikingly decreased from 35.55% in 16S rDNA data to 1.09% in 16S rRNA data.

## Discussion

The PM microbiome in the CSFL fermentation pit produces various flavour compounds such as alcohols, acetic acid, CA. These flavour compounds contribute to the distinct aroma and good taste of the CSFL. The CA, as a key flavour substance of the CSFL, is generally produced from ethanol by anaerobic bacteria (e.g. *C. kluyveri*) via chain elongation pathway (Steinbusch *et al*., 2011; Agler *et al*., 2012; Grootscholten *et al*., 2013). In this study, some key genes involved in chain elongation with ethanol as substrate were found in PM metagenomes, indicating that the PM microbiome possesses metabolic potential for the CA production from ethanol. Besides ethanol, we observed high level of lactate in the PM samples. An excess of lactate was recognized to the increase in ethyl lactate that deteriorates the quality of CSFL. One challenging problem for CSFL manufacturers is how to reduce lactate accumulation during the CSFL production (Tao *et al*., 2016). In this study, metagenomic analysis revealed the presence of key genes responsible for converting lactate to acetyl‐CoA, implying that the PM microbiome owned metabolic potential for the CA production via chain elongation with lactate as energy substrate. This was supported by our previous study (Zhu *et al*., 2015), which showed the PM microbiome could substantially produce CA with lactate as sole energy substrate. Moreover, Kucek *et al*. (2016) reported the conversion of L‐lactate into *n*‐caproate in a continuous fed reactor microbiome. These researches promote us to redefine the roles of lactate in the CSFL fermentation.

In PM metagenomes, most genes related to chain elongation were observed to affiliated mainly to the class *Clostridia* and *Bacilli* in the phylum *Firmicutes*. Particularly, the genus *Clostridium* owned the highest abundance of genes for chain elongation (Table S1). The *Clostridium* is reported to produce CA via chain elongation pathway with ethanol as electron donor (Barker *et al*., 1945; Agler *et al*., 2012; Spirito *et al*., 2014). The abundance of the *Clostridium* is more than 50% of total reads in the metagenome generated from a CA‐producing reactor microbiome; thus, this genus is considered to represent important pools of genes for reverse β oxidation and ethanol oxidation (Agler *et al*., 2012). In this study, the *Clostridium* abundance was 12.15% in PM metagenomes, 1.94% and 1.67% in the 16S rDNA and 16S rRNA libraries respectively. This indicated the active roles of the genus in the PM. Besides the *Clostridium*, the genus *Lactobacillus* was dominant contributor to important genes (*Adh*,* Ald* and *Ldh*) responsible for ethanol oxidation and lactate production, especially *Ldh* (Table S1). The *Lactobacillus* produces lactic acid from sugars by homofermentative metabolism or produces alcohol beside lactic acid by heterofermentative metabolism (Kandler, 1983; Papagianni, 2012). High abundances of *Lactobacillus* were observed in PM metagenomes (3.49%), 16S rDNA (2.33%) and 16S rRNA libraries (19.6%) (Table 3). Highly enriched sequences of *Lactobacillus* in 16S rRNA gene pool indicated its active roles in the PM. This was also supported by high level of lactic acid in the PM (Table 4). In addition, we observed high abundances of methanogens in metagenome (17.92%) and 16S rDNA gene pool (35.55%), but low abundances (1.09%) in the 16S rRNA gene data. This suggested that methanogens were not active in situ. It may attribute to low pH (4.45–5.15) in the PM, that may significantly inhibit methanogenesis when pH is lower than 5.45 (VanKessel and Russell, 1996; Kim *et al*., 2004).

**Table 4:** Chemical properties of the PM

Variable	J_626_	J_663_	J_837_
pH	4.51 ± 0.43	4.65 ± 0.55	4.45 ± 0.03
Moisture (%)	50.98 ± 4.71	47.50 ± 0.54	51.2 ± 3.63
Ethanol (mg g^−1^)	8.03 ± 0.23	7.35 ± 0.58	6.21 ± 0.61
Acetic acid (mg g^−1^)	2.96 ± 0.40	2.10 ± 0.41	2.64 ± 0.62
Lactic acid (mg g^−1^)	18.38 ± 2.8	15.15 ± 4.98	18.31 ± 2.93
Butyric acid (mg g^−1^)^a^	1.53 ± 0.13^a^	0.63 ± 0.02^b^	1.04 ± 0.14^c^
Caproic acid (mg g^−1^)	12.30 ± 0.51	14.37 ± 2.97	17.43 ± 4.14

The data represent mean values ± standard deviation from PM samples (*n* = 3).

a Different letters represent significant differences through pairwise comparison at *P* < 0.05 as determined by ANOVA.

Remarkably, the *Clostridial* cluster IV was most dominant populations (accounting for 42.96% of total reads) in 16S rRNA libraries. Meanwhile, it was abundant in PM metagenomes and 16S rDNA gene pools, making up of 3.86% and 7.35% of total reads. This implied that the *Clostridial* cluster IV was a very active population in the PM. This population predominated in a reactor microbiome that produced CA from lactate, accounting for more than 79% of total abundance (Zhu *et al*., 2015). In this study, CCA analysis revealed the positive relationship between *Clostridial* cluster IV and CA production (Fig. S3). Furthermore, phylogenetic analysis showed the close relationship between abundant *Clostridial* cluster IV OTUs and CA‐producing bacteria (Fig. S4), such as *Clostridium* sp. BS‐1 that produce CA from D‐galactitol (Jeon *et al*., 2010), stain CPB11 (GenBank No., KM454168) and CPB6 (KM454167) that produce CA from lactate. The latter two bacterial strains have been isolated from the PM in our laboratory. To date, little is known about metabolic pathways in the CA‐producing *Clostridial* cluster IV, including key enzymes and genes involved in chain elongation. This might be the reason why not so many genes involved in chain elongation could be matched to the *Clostridial* cluster IV (Table S1). The genus *Caloramator* was abundant in 16S rDNA (3.37%) and 16S rRNA (6.71%) gene pools, but rare in metagenome (0.11%). Some members of the genus are reported to produce acetate, ethanol and lactate from glucose (Ogg and Patel, 2009; Rubiano‐Labrador *et al*., 2013). However, no genes involved in chain elongation were matched to this genus. Thus, the genus members might be important in saccharification and acidogenesis. The genera *Bacteroides, Porphyromonas*and *Sedimentibacter* were also abundant members (> 1%) in 16S rDNA and 16S rRNA gene pools. They were reported to produce succinic acid, propionic acid and alcohols by fermenting starch or glucose (Bryant *et al*., 1958; Kawamura *et al*., 2015; Hu *et al*., 2016), but were not observed to produce CA.

**Figure 6:**
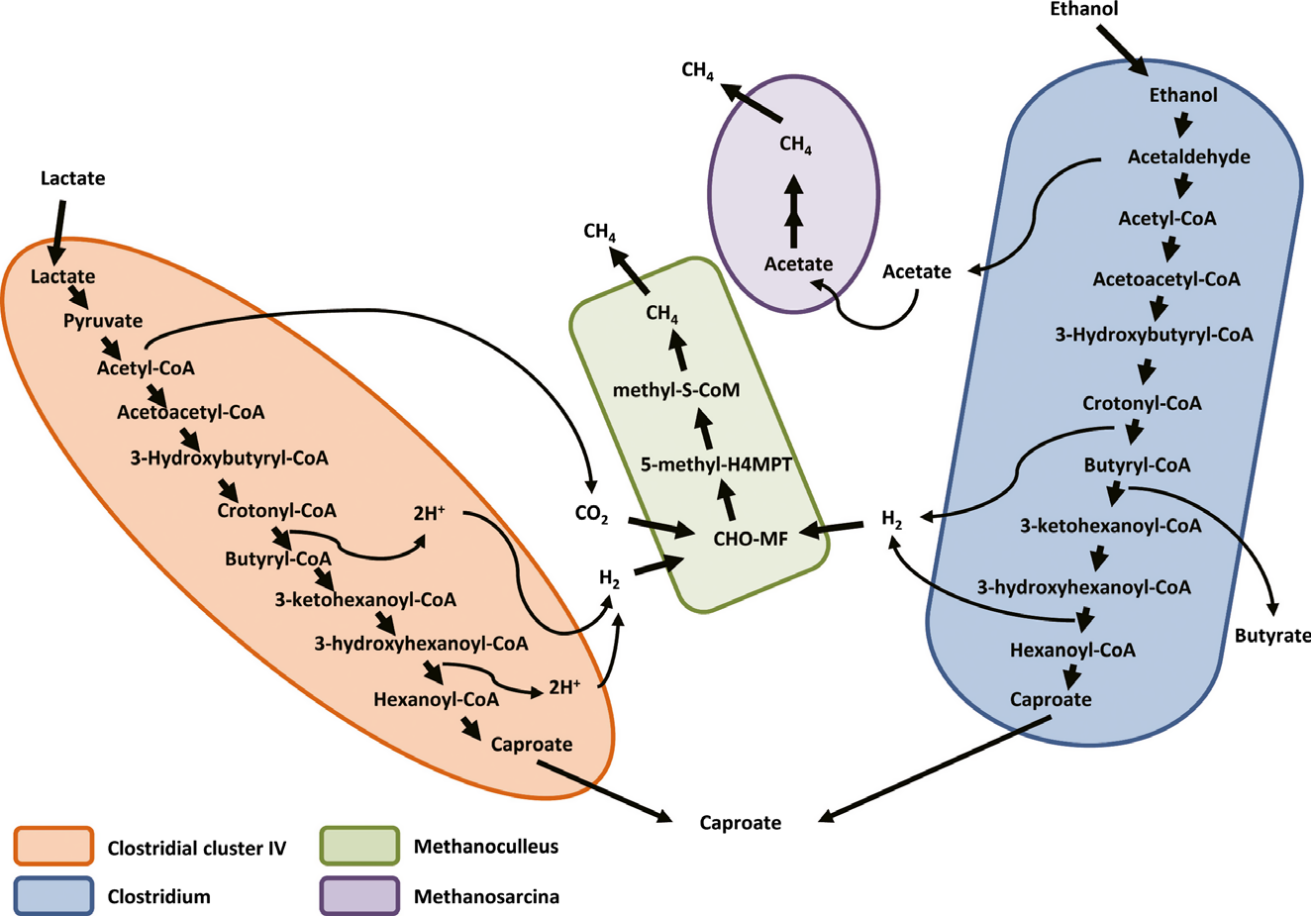
Metabolic reconstructions of the CA‐producing bacteria. The metagenomic data revealed pathways for the CA synthesis with ethanol or lactate as electron donors, and the interspecies hydrogen transfer between CA‐producing bacteria and methanogens.

The *Clostridium* and *Clostridial* cluster IV were observed to be active populations in the PM, which were likely involved in the CA production (Barker *et al*., 1945; Spirito *et al*., 2014; Zhu *et al*., 2015). Therefore, here we preliminarily deduced that the CA production in the PM via chain elongation pathway with lactate or ethanol as electron donor respectively (Fig. 6).Simultaneously, the CA formation is a process of H_2_ and CO_2_ production (Ding *et al*., 2010). The presence of methanogens in PM metagenomes implied that the interspecies hydrogen transfer likely occurred between methanogenesis and chain elongation, which may make CA formation reactions thermodynamically more favourable. However, the interspecies hydrogen transfer could be inhibited by low methanogenic activity due to acidic pH in PM. The relationship between methanogenesis and chain elongation in PM microbiome needs to be investigated further by integrating metatranscriptome and metagenome approaches.

## Materials and methods

### Sampling and chemical property analysis

The PM samples were collected from a famous brewing manufacturer located in Mianzhu city, Sichuan, China. Because the PM microflora keep relative stable between 25 and 50 year (Tao *et al*., 2014), we sampled the pit mud from three CSFL fermentation pits that have been used continuously for 30 years. Pit mud from these pits was acidic (pH 4.45–5.15) and contained abundant ethanol and organic acids such as acetic acid, lactic acid, butyric acid and caproic acid (Table 4). In each fermentation pit, triplicate PM samples were collected from the middle layer of the pit (about 80–100 cm under the ground) and stored at −80 °C until further analyses.

The pH of PM samples was measured by a pH meter in the slurry with 1:5 ratios of samples to deionized water. Moisture was determined with a gravimetric method by drying samples at 85 °C for 48 h immediately after the sampling. For the detection of ethanol and organic acids (e.g. lactic acid, acetic acid and CA) in the PM, the fresh PM was vortex mixed with ultrapure water on a 1:5 (w/v) ratio at room temperature and then centrifuged at 10 000 rpm for 5 min at room temperature. The supernatant was diluted with ultrapure water at a ratio of 1:19 and then filtered through a 0.22‐μm nylon syringe filter (Jinteng Company, China). Ethanol and organic acids were determined by an Agilent 1260 Infinity liquid chromatography system (Agilent Technologies, Palo Alto, CA, USA) equipped with a high‐performance liquid chromatography (HPLC) column Hi‐Plex H (300 × 6.5 mm) at 50 °C, and a differential refraction detector (RID). H_2_SO_4_ (0.005 M) at a flow rate of 0.6 ml min^−1^ was used as the mobile phase.

### DNA extraction and sequencing of metagenomic DNA

Total genomic DNA was extracted using a PowerSoil DNA Isolation Kit (Mo Bio Laboratories, Inc., Carlsbad, CA, USA). The DNA quality was checked by NanoDrop (Thermo Scientific) and agarose gel electrophoresis. Qualified DNAs extracted from triplicate samples of the same pit were pooled. DNA library preparation followed the manufacturer's instruction (Illumina, Inc., San Diego, CA). We constructed three libraries (clone insert size of 200 bp) for sequencing using Illumina HiSeq platform. The base‐calling pipeline (version Illumina Pipeline 1.3) was used to process the raw fluorescence images and call sequences. Raw reads with unknown nucleotides or with low‐quality nucleotides (quality score < 20) were discarded.

### Metagenomic analyses

All raw, unassembled reads were annotated in MG‐RAST (Meyer *et al*., 2008) using Hierarchical Classification subsystems with a maximum e‐value cut‐off of 10^−5^, a minimum 50% identity and 30‐bp alignment length cut‐off. Metagenomic sequences were compared to those corresponding databases. In brief, the GenBank (http://www.ncbi.nlm.nih.gov/GenBank/) taxonomic database was used for analysis of the PM community, and the SEED protein‐coding gene database (http://www.theseed.org) was used for comparison with the putative proteins occurred in the metagenomic data sets. Metabolic pathways were mapped by referring to the Kyoto Encyclopedia of Genes and Genomes (KEGG) (http://www.genome.jp/kegg/) database and chain elongation pathway (Seedorf *et al*., 2008; Spirito *et al*., 2014).

### RNA extraction and cDNA synthesis

To obtain total RNA, PM samples (about 2 g) were processed with an RNA PowerSoil total‐RNA isolation kit (Mo Bio Laboratories, Inc.). The extracted RNA was treated with amplification grade DNase I (Invitrogen, Carlsbad, CA, USA) for 15 min at room temperature according to the manufacturer's instructions and then inactivated by the addition of EDTA at 65 °C for 20 min. The treated sample was concentrated and purified using Amicon Ultra 0.5‐ml centrifugal filters (Millipore Co., Billerica, MA, USA). The cDNA is synthesized with a RevertAid First Strand cDNA Synthesis Kit (Thermo Scientific, Sankt Leon‐Rot, Germany) using random primers provided in the kit, according to the manufacturer's instructions.

### MiSeq sequencing of 16S rDNA and 16S rRNA gene amplicons

The hypervariable V4 region of the 16S rRNA gene was amplified using both DNA and cDNA as templates. DNA and cDNA were diluted to a concentration of 10 ng μl^−1^ for PCR amplification. The universal primers 515F (5′‐GTGCCAGCMGCCGCGGTAA‐3′) and 806R (5′‐GACTACHVGGGTWTCTAAT‐3′) with 10 nt barcodes were used (Yan *et al*., 2015). The PCR was performed according to a previously described method (Yao *et al*., 2014). Each sample was amplified with three technical replicates and then pooled as one sample. The PCR products from different samples were purified through the QIAGEN gel extraction kit (Qiagen, Santa Clarita, CA, USA). Subsequently, purified amplicons were pooled at equimolar concentrations for constructing a PCR amplicon library, according to the Illumina library preparation protocols, and then applied to an Illumina MiSeq system for sequencing (Biobit Biotech Inc. Chengdu, China).

### MiSeq Sequence data analysis

16S rRNA data processing and analysis were performed using QIIME (Caporaso *et al*., 2010). Briefly, the raw sequences were sorted based on the unique barcode of each sample, and the low‐quality sequences with > 0 ambiguous bases, > 6 homopolymers, primer mismatches, average quality scores < 30 and lengths < 200 bp were removed. PCR chimeras were then checked and removed using the UCHIME software (Edgar *et al*., 2011). Each sample was rarefied to the same number of reads (10 000 sequences) from the remaining good‐quality sequences and was clustered into operational taxonomic units (OTUs) at a 97% identity threshold. Taxonomy was assigned using the Ribosomal Database Project classifier. Neighbour‐joining phylogenetic trees of selected sequences were generated with MEGA 6, using a bootstrap method with 1000 replications and a Jukes–Cantor model.

### Statistical analysis

The normality and homoscedasticity of the data were evaluated in SPSS 21 software (IBM SPSS Inc. Chicago, IL, USA). One‐way analysis of variance (ANOVA) performed in SPSS 21 software was used to test the differences in relative abundances of taxonomic units between samples. The difference in chemical property of samples was tested by analysis of variance with the Tukey *post hoc* test. Canonical correspondence analyses (CCA) were performed using CANOCO 5.0 software (Microcomputer Power, Ithaca, NY, USA).

### Sequence deposit

Metagenomic sequences were deposited in MG‐RAST with accession numbers 4579806.3, 4579807.3 and 4579809.3. The original sequence data (16S rRNA gene sequence from DNA and cDNA) are available at the European Nucleotide Archive by Accession PRJEB19933. (http://www.ebi.ac.uk/ena/data/view/PRJEB19933).

## Conflict of interest

None declared.

## Supporting information


**Fig. S1.** The sketch of CSFL fermentation pit.
**Fig. S2.** Predicted metabolic profiles of PM metagenomic datasets related to central carbohydrate metabolism.
**Fig. S3.** Canonical correspondence analyses (CCA) analysis of dominant microbial populations and metabolites.
**Fig. S4.** Neighbor‐joining phylogenetic tree of the microbial representative sequences from the PM 16S ribosomal cRNA data set with the number of representing reads indicated in parentheses. Bootstrap values ≥ 60% of 1000 replicates are indicated at the nodes.
**Table S1.** The percentages of genera matched to the genes encoding enzymes facilitating butyrate/caproate in the PM.
