# Supplementary material for: The functional potential and active populations of the pit mud microbiome for the production of Chinese strong‐flavour liquor
Source: Microb Biotechnol. 2017 Jul 13;10(6):1603–15. doi: 10.1111/1751-7915.12729 (PMC5658580; doi:10.1111/1751-7915.12729)
Supplement: Supplementary file 1 — Fig. S1. The sketch of CSFL fermentation pit. Fig. S2. Predicted metabolic profiles of PM metagenomic datasets related to central carbohydrate metabolism. Fig. S3. Canonical correspondence analyses (CCA) analysis of dominant microbial populations and metabolites. Fig. S4. Neighbor‐joining phylogenetic tree of the microbial representative sequences from the PM 16S ribosomal cRNA data set with the number of representing reads indicated in parentheses. Bootstrap values ≥ 60% of 1000 replicates are indicated at the nodes. Table S1. The percentages of genera matched to the genes encoding enzymes facilitating butyrate/caproate in the PM. [file MBT2-10-1603-s001.pdf]

Supplementary files

The functional potential and active populations of the microbiome in pit mud used for the production of Chinese strong-flavour liquor

Yong Tao[1], Xiang Wang[1], Xiangzhen Li[1*], Na Wei[1], Hong Jin[3], Zhancheng Xu[2], Qinglan Tang[2], Xiaoyu Zhu[1*]

[1] Key Laboratory of Environmental and Applied Microbiology, Chinese Academy of Sciences & Environmental Microbiology Key Laboratory of Sichuan Province, Chengdu Institute of Biology, Chinese Academy of Sciences, Sichuan, 610041, PR China

[2] The National-recognized Enterprise Technology Center, Sichuan Jiannanchun Group Co. Ltd., Mianzhu, Sichuan, 618200, PR China

[3] Chengdu Medical College, Chengdu 610083, China

* To whom correspondence should be addressed. (Tel: +86 28 82890211; Fax: +86 28 82890288; E-mail: zhuxy@cib.ac.cn (XZ) and Lixz@cib.ac.cn (XL)

Key words

Microbiome; Chinese strong-flavour liquor; Pit mud; Metagenomics ; Caproic acid

Chinese strong-flavor liquor brewing process

Chinese strong-flavored liquor (CSFL) is produced by traditional solid-state fermentation method, which has several thousand years of history in Chinese. In brief, fermentation materials (wheat, sorghum and corn) are put into a fermentation pit, in which the entire inner wall is covered with pit mud, which contains various flavor producing microbiomes. For about every 70 days of fermentation period, fermentation mash are taken out for CSFL distilling.

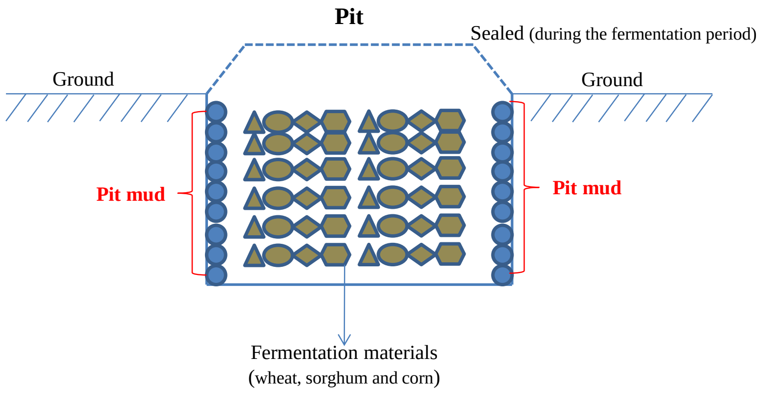

Fig. S1 The sketch of CSFL fermentation pit

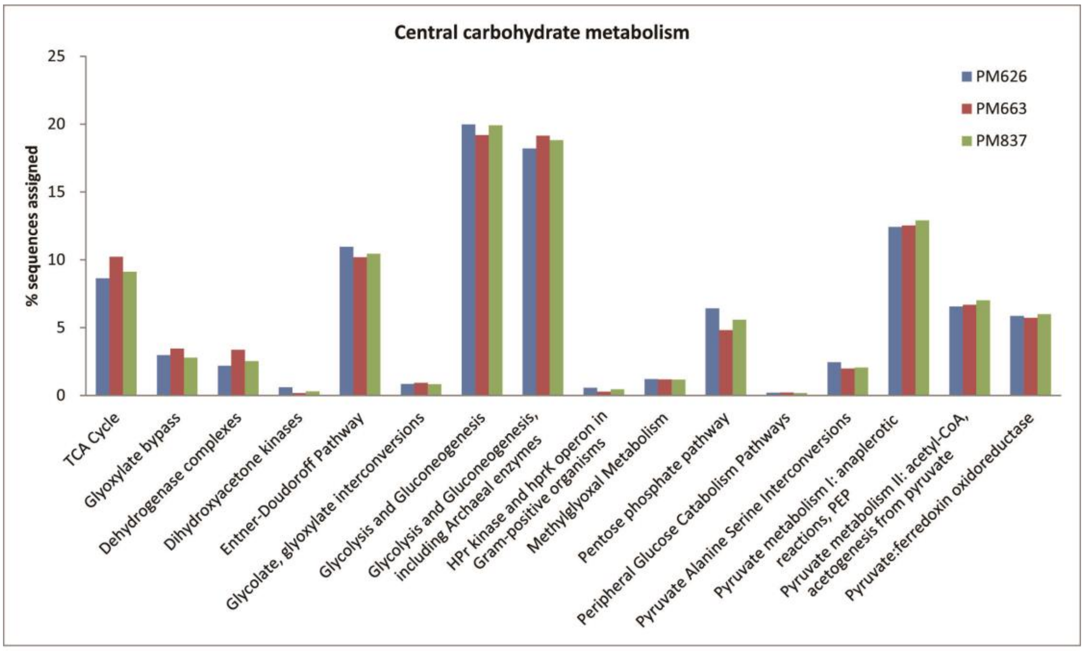

Fig. S2. Predicted metabolic profiles of PM metagenomic datasets

related to central carbohydrate metabolism.

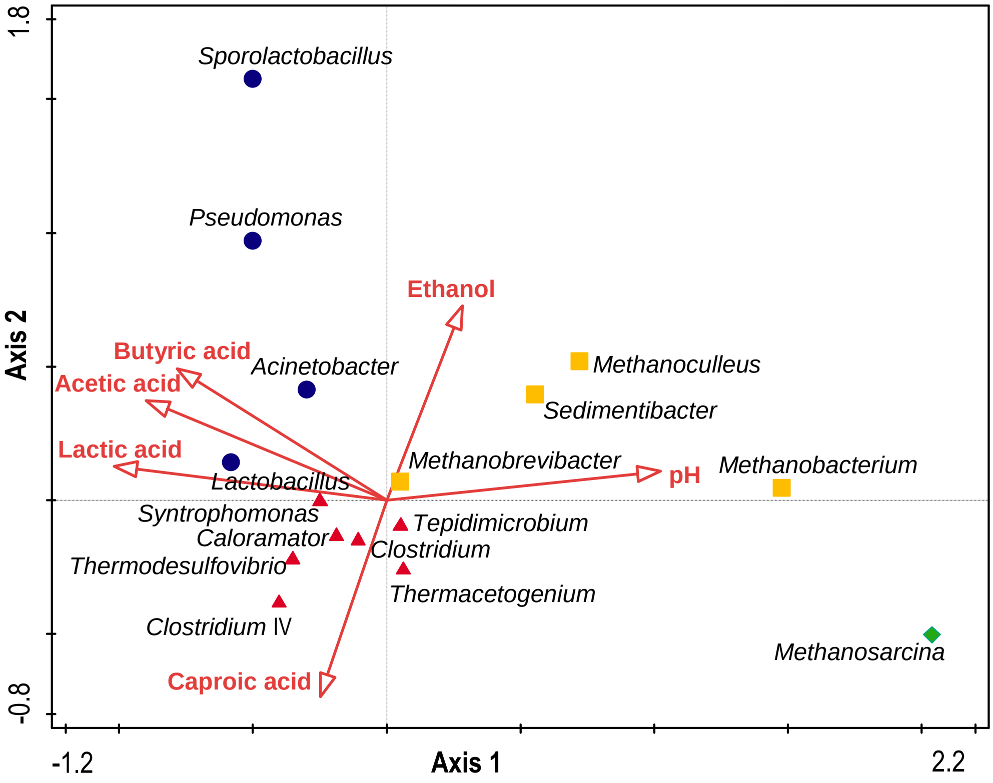

Fig. S3 Canonical correspondence analyses (CCA) analysis of dominant microbial populations and metabolites.

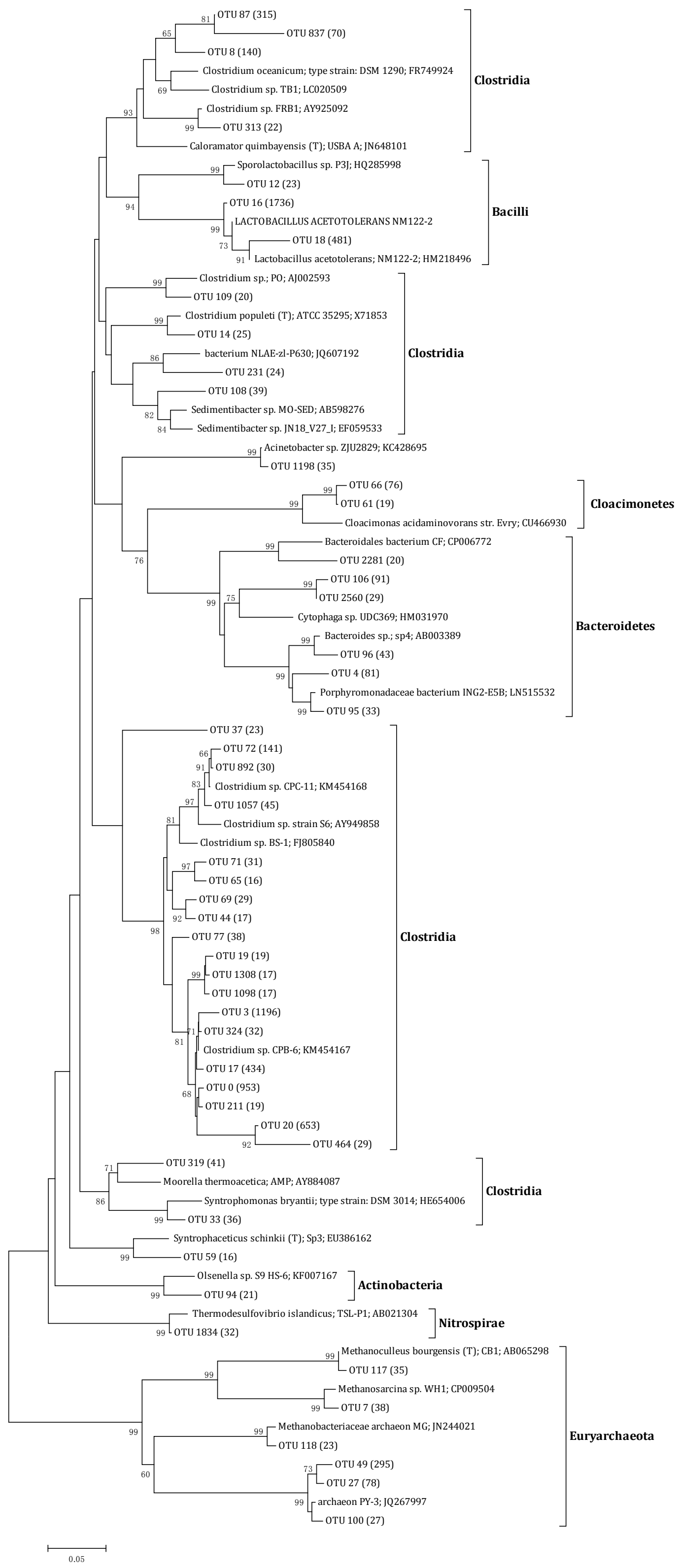

Fig. S4. Neighbor-joining phylogenetic tree of the microbial representative sequences from the PM 16S ribosomal cRNA data set with the number of representing reads indicated in parentheses. Bootstrap values ≧ 60% of 1000 replicates are indicated at the nodes.

Table S1. The percentages of genera matched to the genes encoding enzymes facilitating butyrate/caproate in the PM

Genus/Enzyme/EC	%[a]	Phylum/Class
Alcohol dehydrogenase (EC 1.1.1.1)		
Atopobium	13.2	Actinobacteria
Bacillus	4.7	Bacilli
Lactobacillus	11.8	Bacilli
Clostridium	37.8	Clostridia
Desulfotomaculum	2.0	Clostridia
Dethiosulfovibrio	1.3	Clostridia
Eubacterium	1.2	Clostridia
Halothermothrix	1.5	Clostridia
Thermoanaerobacter	1.4	Clostridia
Elusimicrobium	2.5	Elusimicrobia
Methanosarcina	4.3	Euryarchaeota
Petrotoga	2.3	Thermotogae
Sebaldella	1.0	Fusobacteria
Shewanella	1.3	Proteobacteria
Acetaldehyde dehydrogenase (EC 1.2.1.10)		
Atopobium	15.5	Actinobacteria
Bacillus	5.4	Bacilli
Lactobacillus	13.6	Bacilli
Streptococcus	1.0	Bacilli
Clostridium	43.8	Clostridia
Desulfotomaculum	1.8	Clostridia
Dethiosulfovibrio	1.6	Clostridia
Halothermothrix	1.8	Clostridia
Thermoanaerobacter	1.6	Clostridia
Elusimicrobium	2.9	Elusimicrobia
Sebaldella	1.2	Fusobacteria
Shewanella	1.4	Proteobacteria
Lactate dehydrogenase (EC 1.1.1.27)		
Atopobium	4.4	Actinobacteria
Bifidobacterium	8.7	Bacilli
Enterococcus	1.7	Bacilli
Lactobacillus	41.8	Bacilli
Oenococcus	2.2	Bacilli
Pediococcus	2.0	Bacilli
Caldicellulosiruptor	1.3	Clostridia

Thermoanaerobacter	1.1	Clostridia
Clostridium	27.7	Clostridia
Chloroflexus	1.1	Chloroflexi
Cyanothece	1.2	Cyanobacteria
Pyruvate dehydrogenase complex (EC 1.2.4.1/2.3.1.12)		
Acidothermus	1.5	Actinobacteria
Tropheryma	1.2	Actinobacteria
Xylanimonas	1.1	Actinobacteria
Alicyclobacillus	1.0	Bacilli
Bacillus	2.3	Bacilli
Enterococcus	8.4	Bacilli
Lactobacillus	4.0	Bacilli
Staphylococcus	4.7	Bacilli
Alkaliphilus	20.1	Clostridia
Caldanaerobacter	3.9	Clostridia
Croceibacter	1.3	Bacteroidetes
Polaribacter	1.4	Bacteroidetes
Geobacter	2.7	Proteobacteria
Myxococcus	1.4	Proteobacteria
Acholeplasma	18.1	Tenericutes
Mesoplasma	1.1	Tenericutes
Mycoplasma	5.1	Tenericutes
Candidatus Phytoplasma	2.4	Tenericutes
Sphaerobacter	1.4	Chloroflexi
Acetyl-CoA acetyltransferase (EC 2.3.1.9)		
Lactobacillus	3.4	Bacilli
Staphylococcus	1.0	Bacilli
Streptococcus	2.1	Bacilli
Alkaliphilus	3.4	Clostridia
Anaerococcus	1.5	Clostridia
Caldanaerobacter	1.8	Clostridia
Carboxydothermus	2.7	Clostridia
Clostridium	20.1	Clostridia
Desulfotomaculum	8.5	Clostridia
Eubacterium	5.6	Clostridia
Syntrophomonas	20.7	Clostridia
Desulfatibacillum	1.2	Proteobacteria
Fusobacterium	1.6	Fusobacteria
Brachyspira	2.0	Spirochaetes
Rhodopseudomonas	1.1	Proteobacteria
Syntrophus	2.0	Proteobacteria

3-Hydroxybutyryl-CoA dehydrogenase
(EC 1.1.1.157)

Alkaliphilus	8.0	Clostridia
Caldanaerobacter	6.0	Clostridia
Carboxydothermus	2.5	Clostridia
Clostridium	10.6	Clostridia
Desulfotomaculum	5.9	Clostridia
Eubacterium	1.7	Clostridia
Syntrophomonas	19.0	Clostridia
Atopobium	1.3	Actinobacteria
Fusobacterium	5.7	Fusobacteria
Methanobrevibacter	16.9	Euryarchaeota
Methanosphaera	11.8	Euryarchaeota
Brachyspira	3.6	Spirochaetes

3-Hydroxybutyryl-CoA dehydratase
(EC 4.2.1.55)

Alkaliphilus	3.9	Clostridia
Caldanaerobacter	2.1	Clostridia
Carboxydothermus	1.8	Clostridia
Clostridium	30.7	Clostridia
Desulfotomaculum	4.1	Clostridia
Eubacterium	1.6	Clostridia
Syntrophomonas	32.3	Clostridia
Heliobacterium	2.0	Clostridia
Fusobacterium	9.0	Fusobacteria
Geobacter	1.0	Proteobacteria
Porphyromonas	1.9	Bacteroidetes
Brachyspira	4.1	Spirochaetes

Butyryl-CoA dehydrogenase (EC 1.3.99.2)

Alkaliphilus	8.8	Clostridia
Caldanaerobacter	1.6	Clostridia
Carboxydothermus	4.4	Clostridia
Clostridium	52.0	Clostridia
Eubacterium	1.7	Clostridia
Syntrophomonas	6.2	Clostridia
Natranaerobius	1.8	Clostridia
Fusobacterium	7.3	Fusobacteria
Porphyromonas	3.7	Proteobacteria

Acetyl-CoA:acetoacetyl-CoA
transferase (EC 2.8.3.8)

Bacillus	6.0	Bacilli
Streptococcus	1.1	Bacilli

Candidatus Koribacter	1.2	Acidobacteria
Fusobacterium	6.3	Fusobacteria
Citrobacter	4.6	Proteobacteria
Dinoroseobacter	4.6	Proteobacteria
Escherichia	15.8	Proteobacteria
Haemophilus	21.1	Proteobacteria
Histophilus	3.2	Proteobacteria
Photobacterium	3.3	Proteobacteria
Polynucleobacter	9.5	Proteobacteria
Shigella	13.7	Proteobacteria
Thermosipho	3.8	Thermotogae
Yersinia	3.0	Proteobacteria
Phosphate butyryltransferase (EC 2.3.1.19)		
Bacillus	2.9	Bacilli
Bacteroides	2.9	Bacteroidetes
Alkaliphilus	13.0	Clostridia
Caldanaerobacter	9.5	Clostridia
Clostridium	35.4	Clostridia
Coprothermobacter	4.9	Clostridia
Halothermothrix	2.9	Clostridia
Natranaerobius	1.6	Clostridia
Thermoanaerobacter	5.5	Clostridia
Fervidobacterium	7.5	Thermotogae
Petrotoga	4.0	Thermotogae
Thermosipho	5.2	Thermotogae
Thermanaerovibrio	1.7	Synergistetes
Butyrate Kinase (EC 2.7.2.7)		
Bacillus	5.5	Bacilli
Exiguobacterium	1.1	Bacilli
Geobacillus	1.2	Bacilli
Listeria	1.2	Bacilli
Bacteroides	3.6	Bacteroidetes
Alkaliphilus	12.5	Clostridia
Caldanaerobacter	10.9	Clostridia
Clostridium	35.6	Clostridia
Coprothermobacter	1.3	Clostridia
Thermoanaerobacter	3.1	Clostridia
Natranaerobius	1.3	Clostridia
Geobacter	2.7	Proteobacteria
Pelobacter	1.4	Proteobacteria
Dethiosulfovibrio	1.1	Synergistetes

Thermanaerovibrio	1.1	Synergistetes
Petrotoga	1.1	Thermotogae
Fervidobacterium	3.0	Thermotogae
Thermosipho	2.9	Thermotogae
Thermotoga	1.1	Thermotogae
3-Hydroxy-acyl-CoA dehydrogenase (EC 1.1.1.35)		
Syntrophomonas	28.3	Clostridia
Methanobrevibacter	12.2	Methanobacteria
Methanosphaera	11.6	Methanobacteria
Desulfotomaculum	7.5	Clostridia
Fusobacterium	6.1	Fusobacteria
Clostridium	6.1	Clostridia
Carboxydothermus	5.2	Clostridia
Brachyspira	4.1	Spirochaetia
Porphyromonas	1.8	Bacteroidia
Atopobium	1.7	Actinobacteria
Acyl-CoA hydrolase (EC 3.1.2.20)		
Clostridium	100.0	Clostridia

[a] All data are mean values of PM samples (n=3).
